# Molecular docking analysis of tyrosinase with compounds from poly-herbal formulation for vitiligo treatment

**DOI:** 10.6026/973206300210369

**Published:** 2025-03-31

**Authors:** Karpagambal Ramamoorthy, Manjari Venkatraman, Abarna Balasubramani, Raghavi Marimuthu, Kalaivanan Karuppan, Nithyashree Murugappa

**Affiliations:** 1 Department of Nanju Maruthuvam, National Institute of Siddha, Chennai - 600047, Tamil Nadu, India; 2 Department of Siddha Medicine, Government Primary Health Centre, Tiruvannamalai, Tamil Nadu, India; 3 Department of Siddha Medicine, BSMS - II Professional, Nandha Siddha Medicial College and Hospital, Pitchandipalayam, Erode - 638052, Tamil Nadu, India

**Keywords:** Anti-vitiligo, docking, mookirattai chooranam, siddha formulation, tyrosinase enzyme

## Abstract

Vitiligo is an acquired depigmentary disorder caused by the absence of melanocytes, affecting 0.1% to 2% of the global population, including both adults and children. Therefore, it is of interest to report the molecular docking analysis of tyrosinase (PDB: 1WX3) with compounds from poly-herbal formulation for vitiligo treatment. Analysis shows that the lead bioactive compounds exhibit binding energies ranging from -3.10 Kcal/mol to -7.36 Kcal/mol having 2-6 hydrogen bond interactions with key amino acid residues in the target protein. Beta-sitosterol showed the highest binding affinity (-7.36 Kcal/mol), followed by Orientin (-7.06 Kcal/mol) and other compounds such as masilinic acid, luteolin, glycyrrhizin, corilagin, gallic acid, boeravinone B and trigonelline. Thus, the phytochemicals in the poly-herbal formulation enhance the activity of the tyrosinase enzyme, supporting melanogenesis, making it a potential treatment for vitiligo.

## Background:

Vitiligo is a condition characterized by acquired pigment disorder of the skin andmucous membranes, with a loss of epidermal melanocytes, often accompanied by skin depigmentation and leucoplakia. This condition does not provideitch, pain and physical discomfort but can cause significant psychological stress and ignominy [1]. In India, vitiligo is highly prevalent in Gujarat and Rajasthan up to 8.8%, typically affecting children and adults, with an incidence rate ranging from 0.5% to 2% [2, 3]. A religious myth incorrectly associates vitiligo with sin. Various factors, such as metabolic imbalances, oxidative stress, the production of inflammatory mediators and autoimmune responses, may play a role in the development of this skin condition [4]. Throughout history, herbal remedies have played a vital role in treating skin diseases, offering hope and relief to mankind. Venpadai, Swethakuttam, Venpulli and Venkuttam are synonyms for vitiligo in the Siddha system of medicine. In Siddha literature, Siddhar Yugimuni mentioned skin diseases as Kuttam and classified them into 18 types, Venkuttam or Swetha Kuttam (vitiligo) is one among them [5]. The treatment strategies for vitiligo couldn't effectively encourage total repigmentation with durable results and prevent recurrence. Repigmentation can be treated with a variety of approaches, including oral systemic drugs, calcineurin inhibitors, topical corticosteroids and narrowband ultraviolet B (NB-UVB) light therapy and Janus kinase inhibitors can cause side effects including erythema, pruritis, hyperpigmentation upper respiratory infections, weight gain, arthralgia, mild elevation of lipid levels and transient acne [6, 7]. In Siddha, vitiligo can be treated through many internal and external medicines. One of the polyherbal formulations is "Mookirattai chooranam" composed of 4 ingredients *Terminalia chebula* fruit, *Cassia roxburghii* root, *Boerrhavia diffusa* root and *Abrus precatorius* root indicated for kuttam [8]. The activity of tyrosinase to promote melanogenesis (which is the process of producing melanin pigment in melanocytes) is known [9]. Therefore, it is of interest to report the molecular docking analysis of tyrosinase (PDB: 1WX3) with compounds from poly-herbal formulation for vitiligo treatment.

## Materials and Methods:

## Tyrosinase protein target structure:

Figure 1 (see PDF) represents the crystalline structure of the target protein tyrosinase with PDB 1WX3, derived from the protein data bank and it is processed for protein clean-up and the addition of missing hydrogen atoms.

## Ligand molecule structures:

The ligand molecules were selected by literature search and the required molecules were retrieved from the PubChem database, represented in Table 1. 2D and 3D structures of ligands were in Figure 2 (see PDF).

## Molecular docking analysis:

The auto dock program analysed various lead molecule orientations regarding the target protein and the best dock pose was chosen based on the interaction study results (Figure 3(a-i) - see PDF) [10]. Auto dock 4 was used to make docking calculations. The ligand atoms are added with Gustier partial charges. Non-polar hydrogen atoms were merged and rotatable bonds were defined. Docking was carried out for phytochemical constituents derived from literature against tyrosinase enzyme PDB 1WX3. With the use of auto dock tools, necessary hydrogen atoms, Kollman unified atom type charges and solvation parameters were added. Using the auto grid program, the Affinity of maps and Spacing was generated as xx Å grid points and 0.375 Å respectively. The Vander Waals and electrostatic terms were computed using auto dock parameter set- and distance-dependent dielectric functions. The Lamarckian genetic algorithm (LGA) and the Solis and Wets local search approach were used to simulate docking. The ligand molecules' initial positions, orientations and torsion were determined randomly. During docking, all rotatable torsions were freed. Each docking experiment was constructed from two separate runs, each programmed to end after a maximum of 250000 energy assessments. The population was limited to 150 people. A translational step of 0.2, a quaternion step of 5 and a torsion step of 5 were all used during the study [11, 12-13].

## Results and Discussion:

Vitiligo patients experience significant negative effects on their physical and mental health, loss of skin photo-protection weakened immunity and deterioration in the quality of life that is associated with the disease development at a young age [14]. The therapeutic goal for vitiligo is to depigmentation of skin colour and to stop the spread of depigmentation of the skin. The action of tyrosinase enzyme is to improve melanogenesis which induces melanin pigment in depigmented skin. Tyrosinase is a sensitive enzyme, responsible for stimulating the production of melanin pigment and is a principal auto-antigen of autoimmune vitiligo [15]. Tyrosinase assay and comet assay on 21 patients revealed that tyrosinase activity was lower in lesional vitiligo than in non-lesional vitiligo patients. Similarly, increased accumulation of oxidative stress leads to decreased tyrosinase activity [16]. Thus, by increasing the activity of tyrosinase, melanogenesis is promoted which helps to retrieve the skin color.

Evaluation of "Mookirattai chooranam" with 9 lead bioactive compounds like Gallic acid, Corilagin, Masilinic acid from *Terminalia chebula*, [17] Beta-sitosterol from *Cassia roxburghii*, [18] Boeravinone B from *Boerrhavia diffusa* [19] and Luteolin, Orientin, Glycyrrhizin, Trigonelline from *Abrus precatorius* [20] were retrieved by literature review and given in Table 1. The lead bioactive constituents docking score with tyrosinase exhibit binding energy ranges from -3.10 Kcal/mol to -7.36Kcal/mol and possess 2-6 interactions with the core target amino acid residue His38, His54, His63, His 190, His194 and His216 present in the active site of tyrosinase enzyme (Table 2 and Table 3) (Figure 2 - (see PDF) and Figure 3 - (see PDF)). It was found that beta-sitosterol showed the highest binding affinity of -7.36Kcal/mol, Orientin showed second highest binding affinity of-7.06Kcal/mol to the amino acid residues His54, His 64, His 190, His 194 followed by masilinic acid, luteolin, glycyrrhizin, corilagin, gallic acid, boeravinone B and trigonelline with binding energies of -6.62Kcal/mol, -5.68 Kcal/mol, -4.79 Kcal/mol, -4.25 Kcal/mol. -4.21 Kcal/mol, -4.13 Kcal/mol and -3.10 Kcal/mol respectively in the descending order of magnitude. Thus, beta-sitosterol possessing the maximum binding energy with a molecular weight of 414.718 g/mol, a log P value of 10.482 (est.) and one hydrogen bond donor and acceptor, beta-sitosterol satisfies Lipinski's rule of 5. Lee *et al.* 1994 conducted a rodent study that suggested that beta-sitosterol may be effective in the treatment of vitiligo condition [21].

Masilinic acid interacts with 1 amino acid residue His 194, gallic acid, corilagin and trigonelline shared 2 active amino acid sites in common. Boeravinone B, luteolin shared 3 active amino acid sites. Orientin, Beta-sitosterol and glycyrrhizin possess 4, 5, 6 maximum interactions with the active site of the target core amino acid residues of tyrosinase enzyme. While considering the interactions, Glycyrrhizin possesses the highest interactions with all the 6-target core amino acid residues. The amino acid 54 and 190 binds with a maximum of 8 phytochemicals and the amino acid 194 binds with 6phytochemicals present in the formulation. The 9 lead bioactive compounds of "Mookirattai chooranam" reveals a maximum of 2-6 interactions with the core active amino acids residues present in the target tyrosinase. The graphical representation of this study is in Figure 4 - (see PDF). Research studies on Gallic acid from *Terminalia chebula* by Manosroi *et al.* 2011 proved that gallic acid acts as an antioxidant agent used to treat vitiligo conditions [22]. *In-vitro* study on luteolin promotes melanogenesis by activating tyrosinase enzyme [23]. According to Meena *et al.* the Insilco anti-vitiligo activity of Glycyrrhizin exhibits a good docking score on IL-17 inhibitors and possesses good antioxidant potential [24].

## Conclusion:

Molecular docking analysis shows that the lead bioactive compounds exhibit binding energies ranging from -3.10 Kcal/mol to -7.36 Kcal/mol having 2-6 hydrogen bond interactions with key amino acid residues in the target protein. Beta-sitosterol showed the highest binding affinity (-7.36 Kcal/mol), followed by Orientin (-7.06 Kcal/mol) and other compounds such as masilinic acid, luteolin, glycyrrhizin, corilagin, gallic acid, boeravinone B and trigonelline. Thus, the phytochemicals in the poly-herbal formulation enhance the activity of the tyrosinase enzyme, supporting melanogenesis, making it a potential treatment for vitiligo.

## Author's contribution:

Karpagambal Ramamoorthy, Manjari Venkatraman and Abarna Balasubramani are did the study, analysis interpretations of data, report writing and formatting. Raghavi Marimuthu, Kalaivanan Karuppan and Nithyashree Murugappa are did literature review and formatting.

## Figures and Tables

**Table 1:** Properties of the ligand compounds selected for docking analysis

**Medicinal Drug**	**Compound**	**Molecular formula**	**Molar weight g/mol**	**H Bond Donor**	**H Bond Acceptor**	**Rotatable bonds**
*Terminalia chebula*	Gallic acid	C_7_H_6_O_5_	170.12g/mol	4	5	1
	Corilagin	C_27_H_22_O_18_	634.5 g/mol	11	18	3
	Maslinic acid	C_30_H_48_O_4_	472.7 g/mol	3	4	1
*Cassia roxburghii*	Beta-Sitosterol	C_29_H_50_O	414.718g/mol	1	1	6
*Boerhavia diffusa*	Boeravinone B	C_17_H_12_O_6_	312.27 g/mol	3	6	0
*Abrus precatoris*	Luteolin	C_15_H_10_O_6_	286.24g/mol	4	6	1
	Orientin	C_21_H_20_O_11_	448.4 g/mol	8	11	3
	Glycyrrhizin	C_42_H_62_O_16_	822.9 g/mol	8	16	7
	Trigonelline	C_7_H_7_NO_2_	137.14 g/mol	0	2	0

**Table 2:** Summary of the molecular docking studies of compounds with tyrosinase (1WX3)

**Compound**	**Est. Free Energy of Binding**	**Est. Inhibition Constant, Ki**	**Electrostatic Energy**	**Total Intermolec. Energy**	**Interact. Surface**
Gallic acid	-4.21 kcal/mol	817.05 uM	-0.28 kcal/mol	-3.76 kcal/mol	376.525
Corilagin	-4.25 kcal/mol	763.52 uM	-0.35 kcal/mol	-3.83 kcal/mol	789.353
Maslinic acid	-6.61 kcal/mol	14.18 uM	-0.09 kcal/mol	-6.33 kcal/mol	724.114
Beta-Sitosterol	-7.36 kcal/mol	4.02 uM	-0.05 kcal/mol	-8.44 kcal/mol	697.43
Boeravinone B	-4.13 kcal/mol	941.43 uM	-0.25 kcal/mol	-5.02 kcal/mol	587.052
Luteolin	-5.68 kcal/mol	68.93 uM	-0.30 kcal/mol	-5.46 kcal/mol	596.603
Orientin	-7.06 kcal/mol	6.73 uM	-0.19 kcal/mol	-5.46 kcal/mol	688.708
Glycyrrhizin	-4.79 kcal/mol	307.83 uM	-0.24 kcal/mol	-4.51 kcal/mol	711.177
Trigonelline	-3.10 kcal/mol	5.34 uM	-0.61 kcal/mol	-3.40 kcal/mol	381.745

**Table 3:** Amino acid residue interaction of lead compounds with tyrosinase (1WX3) HIS38, HIS54, HIS63, HIS 190, HIS194 and HIS216

**Compound**	**Interaction Amino acid Residues**																
Gallic acid	2	42	45	54	55	182	184	190	191								
		ILE	ASP	HIS	ARG	GLU	TRP	HIS	ASN								
Corilagin	3	42	54	55	182	184	188	190	191	194	195						
		ILE	HIS	ARG	GLU	TRP	ASN	HIS	ASN	HIS	VAL						
Maslinic acid	1	42	184	188	191	194	195	206									
		ILE	TRP	ASN	ASN	HIS	VAL	SER									
Beta-Sitosterol	5	38	42	54	184	190	191	194	195	206	216						
		HIS	ILE	HIS	TRP	HIS	ASN	HIS	VAL	SER	HIS						
Boeravinone B	3	42	54	55	182	184	190	191	194	195							
		ILE	HIS	ARG	GLU	TRP	HIS	ASN	HIS	VAL							
Luteolin	3	42	54	55	182	184	188	190	191	194	195						
		ILE	HIS	ARG	GLU	TRP	ASN	HIS	ASN	HIS	VAL						
Orientin	4	42	54	55	59	63	182	184	190	191	194	195	202				
		ILE	HIS	ARG	PHE	HIS	GLU	TRP	HIS	ASN	HIS	VAL	ALA				
Glycyrrhizin	6	38	42	54	55	59	63	182	184	188	190	191	192	194	195	206	216
		HIS	ILE	HIS	ARG	PHE	HIS	GLU	TRP	ASN	HIS	ASN	ARG	HIS	VAL	SER	HIS
Trigonelline	2	42	54	55	182	184	190	191									
		ILE	HIS	ARG	GLU	TRP	HIS	ASN									
